# Application of Machine Learning Method in Genomics and Proteomics

**DOI:** 10.1155/2015/914780

**Published:** 2015-04-19

**Authors:** Hao Lin, Wei Chen, Ramu Anandakrishnan, Dariusz Plewczynski

**Affiliations:** ^1^Center of Bioinformatics, School of Life Science and Technology, University of Electronic Science and Technology of China, Chengdu 610054, China; ^2^Department of Physics, Center for Genomics and Computational Biology, College of Sciences, Hebei United University, Tangshan 063000, China; ^3^Department of Computer Science, Laboratory for Theoretical and Computational Molecular Biophysics, Virginia Tech, Blacksburg, VA 24060, USA; ^4^Center of New Technologies, University of Warsaw, 02097 Warszawa, Poland

With the avalanche of genomic and proteomic data generated in the postgenomic age, it is highly desirable to develop automated methods for rapidly and effectively analyzing and predicting the structure, function, and other properties of DNA and protein. The machine learning methods have become an important strategy for the discovery of potential knowledge in genomics and proteomics. Researches in recent years have shown tremendous advances in the properties prediction of DNA fragments and protein sequences by various pattern recognition methods. These techniques provide economical and timesaving solutions for identifying the properties of DNA and protein. This special issue was hosted for the recent development of the application of machine learning methods in genomics and proteomics.

In this special issue, five works focused on the protein classification. How to extract key features from a protein was a key step in the discrimination of protein class. B. Liu et al. proposed to use Position-Specific Score Matrix (PSSM) and Accessible Surface Area (ASA) to formulate protein samples. The hidden Markov support vector machine (HM-SVM) was employed to predict protein binding site. Simulation in fivefold cross-validation on a benchmark dataset including 1124 protein chains showed that their method is more accurate for protein binding site prediction than some state-of-the art methods. This method can also be applied in DNA binding site, vitamin binding site, and posttranslational modification of proteins.

Based on chemical shift (CS) information derived from nuclear magnetic resonance (NMR), F. Yonge proposed a novel feature to predict protein supersecondary structures. The quadratic discriminant (QD) analysis was selected as the prediction algorithm. Overall accuracy in threefold cross-validation is 77.3% for predicting four types of supersecondary structures. According to the concept of pseudo amino acids, G.-L. Fan et al. proposed the average chemical shifts (ACS) composition and established an online webserver called acACS which was calculated from average chemical shift information and protein secondary structure. By using SVM as the classification algorithm, the acACS was used in the discrimination between acidic and alkaline enzymes and between bioluminescent and nonbioluminescent proteins. Encouraging results were achieved. The protein secondary structure, structure class, and disorder region can be predicted using the AC-based method.

L. Nanni et al. proposed to combine different features to improve protein prediction. These features include amino acids composition, PSSM, and substitution matrix representation (SMR). Each feature is used to train a separate SVM. Total of 15 benchmark datasets were used to evaluate the performance of their proposed method. Comparative results show that the PSSM always produces good accuracies. However, no single descriptor is superior to all others across all test datasets. The major contribution in this paper is to propose an ensemble of classifiers for sequence-based protein classification.

H. Lin et al. briefly reviewed the development of ion channel prediction using machine learning method. They initially introduced how to construct a valid and objective benchmark dataset to train and test the predictor. Subsequently, the mathematical descriptors were presented to formulate the ion channel sequences. Moreover, two feature selection techniques on how to optimize feature set were described. Finally, the support vector machine was suggested performing classification. The methods introduced in that review can be generalized into other protein prediction fields as well.

The paper from P. Feng et al. was the unique work focused on DNA prediction using machine learning method. They proposed a novel descriptor called pseudo K-tuple nucleotide composition (PseKNC) to formulate the DNA sequences. The feature is calculated from K-tuple nucleotide composition and the structural correlation of DNA dinucleotides. Subsequently, the SVM was used to predict DNase I hypersensitive sites. The jackknife cross-validated accuracy is 83%, which is competitive with that of the existing method. This new descriptor can also be widely used in DNA regulatory elements prediction.

*Hao Lin* *Hao Lin* *Wei Chen* *Wei Chen* *Ramu Anandakrishnan* *Ramu Anandakrishnan* *Dariusz Plewczynski* *Dariusz Plewczynski*

